# Climate Change and Vector-borne Diseases: An Economic Impact Analysis of Malaria in Africa

**DOI:** 10.3390/ijerph8030913

**Published:** 2011-03-23

**Authors:** Aklesso Egbendewe-Mondzozo, Mark Musumba, Bruce A. McCarl, Ximing Wu

**Affiliations:** 1 Department of Agricultural, Food, and Resource Economics, Michigan State University, 86 Agriculture Hall, East Lansing, MI 48824, USA; 2 Department of Agricultural Economics, Texas A&M University, 2124 TAMU College Station, TX 77843, USA; E-Mails: mmusumba@vprmail.tamu.edu (M.M.); brucemccarl@gmail.com (B.A.M.); wuximing05@gmail.com (X.W.)

**Keywords:** malaria and climate change, semi-parametric modeling, cost of malaria treatment

## Abstract

A semi-parametric econometric model is used to study the relationship between malaria cases and climatic factors in 25 African countries. Results show that a marginal change in temperature and precipitation levels would lead to a significant change in the number of malaria cases for most countries by the end of the century. Consistent with the existing biophysical malaria model results, the projected effects of climate change are mixed. Our model projects that some countries will see an increase in malaria cases but others will see a decrease. We estimate projected malaria inpatient and outpatient treatment costs as a proportion of annual 2000 health expenditures per 1,000 people. We found that even under minimal climate change scenario, some countries may see their inpatient treatment cost of malaria increase more than 20%.

## 1. Introduction

Vector-borne diseases are among the diseases that have been linked with climate change [1]. Malaria is probably the deadliest climate sensitive vector-borne disease [2]. In 2008, globally there were an estimated 243 million malaria cases with 863,000 deaths. 89% of the reported deaths were in Africa. Most of the deaths were among children under 5 years’ old and pregnant women. The annual economic costs of malaria in Africa in terms of foregone production have been estimated to be about US $12 billion [3]. However, that estimate is likely low as it neglects costs of treatment, loss of life, and lifelong disabilities that often result from childhood infections. Goodman *et al.* [4] estimates that the malaria burden in Africa is about 10% of the region’s total disease burden in terms of disability adjusted life years (DALY). HIV/AIDS is also a factor as it weakens the immune system increasing vulnerability to malaria infection and there are an estimated 25 million infected people. Together, HIV/AIDS and malaria cause more than 4 million deaths per year [5]. Consequently malaria is a critical public health issue in Africa.

A number of researchers have investigated the relationship between malaria and climatic factors as reviewed in [1] and [6]. Zhou *et al.* [7] and Wandiga *et al.* [8] work on African highlands and indicate that climatic factors such as temperature and precipitation play an important role in explaining the variation in malaria cases but these studies were conducted on a small scale (in a specific region of a country) creating a need for larger scale investigation. The role of climate change in the increase of malaria incidence in African highlands has particularly been controversial [9,10] but it is difficult to totally deny the fact that climatic factors play an important role on malaria incidence [11,12]. Most of the economic analyses have been oriented toward cost effectiveness analysis of anti-malaria drugs [3,13,14] and available treatment options [4]. Goodman *et al.* [15] reviewed literature on the measurement of the economic impact of malaria in sub-Saharan Africa and concluded that the few available studies are less reliable and there is a need of more sophisticated research in the area. A number of biophysical research models [16–18] have been developed but do not include economic impact assessment.

This paper reports on a larger scale econometric study of the relationship between climatic factors and reported malaria cases using data from 25 African countries from 1990 to 2000. In turn we examine the potential effect of climate change using IPCC temperature and precipitation projections. This research differs from the existing literature in several noteworthy ways; (1) many relevant socio-economic factors are accounted for in modeling malaria-climate relationship in order to separate socio-economic factors impact from pure climate factors impact, (2) a semi-parametric specification is estimated and tested that does not assume a priori knowledge of the functional relationship between climatic factors and malaria prevalence, (3) more African countries are included in the study and (4) a model based projection of the malaria treatment cost increase/decrease due to climate change by the end of the century is given.

## 2. Malaria and Its Link to Climate: An Overview

Malaria is transmitted by mosquitos carrying malaria parasites. Malaria’s distribution depends on the availability and productivity of mosquito breeding habitat. The availability of the breeding habitat is related to stagnant water that remains after rainfall while productivity of the breeding habitat is a function of the ambient temperature [12,19]. Rainfall rises the abundance of the breeding habitat while higher temperature increases the malaria risk by shortening the malaria parasites development-cycle [20]. The average life span of a mosquito carrying malaria parasites is about 21 days. It takes 19 days for the malaria parasite to mature inside the mosquito at 22 degrees Celsius and 8 days to mature at 30 degrees Celsius. Apart from the African highlands and the farthest southern and northern African regions, the annual mean temperature on the African continent is above 25 degrees Celsius [19]. Therefore, the projected increase in mean temperature of about 1.4 to 5.8 degrees Celsius under climate change [21] may result in a faster parasite development and a potentially higher incidence of malaria.

Recent studies have attempted to estimate the impact of climate change on malaria prevalence. Ebi *et al.* [22] studied climate suitability for stable malaria transmission in Zimbabwe under various climate change scenarios based on MARA/ARMA (Mapping Malaria Risk in Africa/Atlas du Risque de la Malaria en Afrique) decision rules. They indicate that changes in temperature and precipitation could alter the geographic distribution of malaria in Zimbabwe, with previously unsuitable regions with dense human populations becoming suitable. Thomson *et al.* [23] investigated the effect of rainfall and sea surface temperature on malaria incidence in Botswana finding that variability in rainfall and sea temperature accounts for more than two-thirds of the inter-annual variability in malaria incidence. Ndiaye *et al.* [24] studied relationships between climate variability and malaria mortality in a small region of Senegal from 1984 to 1996. They found that 89.1% of the malaria mortality was observed in August and December which are relatively rainy and warmer months. They found high correlation between rainfall variability and malaria mortality but did not find significant correlation with temperature and humidity variability. Jaenisch and Patz [25] find that malaria prevalence is sensitive to short-term fluctuations in temperature and rainfalls in a way that extreme precipitation events could wash away anopheles mosquito larvae from breeding sites and lead to reduced incidence. This argument means that the relationship between malaria cases and climatic factors is of a nonlinear nature and using linear functions to study such relationship will be misleading. Wandiga *et al.* [8] found significant correlation between malaria cases and climatic factors in Uganda but implicitly assumed a linear relationship [25]. Zhou *et al.* [7] pursued a nonlinear relationship study and found that climatic factors were the driving forces behind malaria’s resurgence in highlands of Africa explaining 65 to 81 percent of the variation in number of cases. They also found a statistically significant synergy effect between temperature and precipitation in determining the number of malaria cases in all the regions studied.

The study in this paper builds on these previous studies to improve our understanding of the climate-malaria relationship and the potential economic consequences from climate change. We employ socio-economic variables to study how they might affect malaria prevalence along with temperature and precipitation. Some of these variables may exhibit linear relationships while others might involve nonlinearities. To accommodate this we use semi-parametric methods that allow nonlinearity without assuming a priori knowledge of the functional forms governing the process [10,26–28]. Also, semi-parametric methods offer a way to test the validity of the non-linear relationship between climate and malaria prevalence claimed in previous literature [7,23,25] using a nonparametric statistical test [27].

To carry out this study we will examine malaria incidence in 25 African countries for which data were available (Algeria, Benin, Botswana, Burkina, Burundi, Central African Republic, Chad, Cote D’Ivoire, Djibouti, Egypt, Ethiopia, Ghana, Guinea, Malawi, Mali, Mauritania, Morocco, Niger, Rwanda, South Africa, Sudan, Togo, Uganda, Tanzania, Zimbabwe).To do this we use the World Health Organization (WHO) data on disease incidence from 1990 to 2000 plus associated temperature and precipitation data while controlling for socio-economic confounding factors. Several African countries do not regularly report their national malaria incidence data to the WHO and those with missing data and outliers were not included.

## 3. Model Specifications

The basic data set is in the form of a panel with repeated observations at multiple sites. To carry out the statistical analysis a semi-parametric panel model is specified and estimated [28–30]. Such a model allows us to simultaneously handle nonlinearity in the relationships along with the effects of countries and time (years). Pooling the data across countries and years allows us to capture the likely impact that we might expect to see in a longer, but unavailable, time series for the individual countries. The estimated coefficients give information on the relationship between observed malaria cases, temperature and precipitation. The theoretical model follows the analysis in Li and Racine as well as Tseng *et al.* [28,31] and is specified as follows:

(1) $$y_{\text{it}}=f(X_{\text{it}})+\beta Z_{\text{it}}+\alpha_{i}+u_{\text{it}},\;\;t=1,\;\ldots ,\;T\;\text{and}\;i=1,\;\ldots ,N$$

where *y_it_* is the natural log of the number of reported malaria cases per 1,000 people in country *i* at time period *t; X_it_* is a vector of climate variables that includes temperature, precipitation and a measure of climate variability; *Z_it_* is a vector of socio-economic control variables that includes population density, per capita gross domestic product, inequality index, per capita healthcare expenditure and number of hospital beds per 1,000 people; *α_i_* are unobserved individual country effects and *u_it_* is an idiosyncratic error term. The function *f*, the coefficients β and the unobserved country effects *α_i_* are all parameters to be estimated. Note that climate variables are assumed to affect the number of malaria cases per 1,000 people through an unknown function *f* to be estimated while the socio-economic variables affect malaria cases linearly.

## 4. Model Estimation

The estimation method follows Robinson’s [32] two step estimation procedure and is as follows:

**Step1:** Express the conditional expectation of malaria cases per 1,000 people (*y_it_*) with respect to the climate variables (*X_it_*) as follows:

(2) $$E(y_{\text{it}}|X_{\text{it}})=f(X_{\text{it}})+\beta E(Z_{\text{it}}|X_{\text{it}})$$

where the nonlinear variables *X_it_* are assumed to be uncorrelated with the individual country effects and the idiosyncratic error terms. That is, climatic factors are uncorrelated to unobservable items that affect a particular country’s disease prevalence (e.g., poor environmental conditions, drug resistance) and the statistical error in fitting the model. This assumption allows consistent estimation of the linear parameters of the model. Note also that a measurement error on the number of cases is inevitable as many cases of malaria in remote area are unreported. However, it is well known in the econometric literature [33] that these measurement errors will not affect the consistency of our estimated coefficients.

By subtraction of (2) from (1) we have:

(3) $$y_{\text{it}}-E(y_{\text{it}}|X_{\text{it}})=\beta [Z_{\text{it}}-E(Z_{\text{it}}|X_{\text{it}})]+\alpha_{i}+u_{\text{it}}$$

The conditional expectations are estimated using non-parametric kernel regression methods such that *ỹ_it_* = *y_it_* – *Ê* (*y_it_*|*X_it_*), *Z̃_it_* = [*Z_it_* – *Ê*(*Z_it_*|*X_it_*)] and *Ê*(.|.)is a kernel estimator [28]. Equation (3) is then transformed into a well-known linear form:

(4) $${\tilde{y}}_{\text{it}}=\beta {\tilde{Z}}_{\text{it}}+\alpha_{i}+u_{\text{it}}$$

At this point the estimated values of the parameter *β̂* are obtained using panel data specification estimation methods [34]. Here fixed or random effects panel data econometrics specifications could be applied. The fixed effects specification allows a possible correlation between the variable *Z̃_it_* and the country effects *α_i_*. The random effects specification assumes independence between *Z̃_it_* and the country effects *α_i_*. Since the independence between the individual unobserved effects and the regressors cannot be established ex ante, we use a Hausman specification test [35,36] to test the suitability of the fixed effects assumption. In this test the null hypothesis is that the individual unobserved effects and the regressors are uncorrelated (*H*_0_: *E*(*α_i_*|*Z̃_it_*) = 0).

**Step 2:** Given the estimates of *β̂*, then estimate the function using the relation

(5) $$y_{\text{it}}-\hat{\beta }Z_{\text{it}}=f(X_{\text{it}})+\alpha_{i}+u_{\text{it}}$$

By writing *y_it_* – *β̂Z_it_* = *y̿_it_*, we have the non-parametric form:

(6) $${\bar{\bar{y}}}_{\text{it}}=f(X_{\text{it}})+\alpha_{i}+u_{\text{it}}$$

and is *f* estimated locally minimizing $\sum_{i}\sum_{t}{\left[{\bar{\bar{y}}}_{\text{it}}=f(X_{\text{it}})+\alpha_{i}+u_{\text{it}}\right]}^{2}K\left(\frac{X_{\text{it}}-x}{h}\right)$, with *K*(.) being a kernel density function. Ullah and Mundra [37] show that by expressing *f* as a smooth coefficient function such that *f* (*X_it_*) = *V′_it_θ* (*X_it_*), where *V_it_* = (1, *X_it_*), we can estimate *θ̂*(*X_it_*) as:

(7) $$\hat{\theta }(X_{\text{it}})={\left[\sum_{i}\sum_{t}V_{\text{it}}^{*}V_{\text{it}}^{*\prime }K\left(\frac{X_{\text{it}}-x}{h}\right)\right]}^{-1}\left[\sum_{i}\sum_{t}V_{\text{it}}^{*}{\bar{\bar{y}}}_{\text{it}}^{*}K\left(\frac{X_{\text{it}}-x}{h}\right)\right]$$

where (*) implies that local estimation using kernel estimator on conditional expectations is applied. This specification takes into account the fact that climate variability can cause a jump in the number of malaria cases or change the rate of growth in the malaria cases. This particular specification also reduces the dimensionality of the nonparametric function *f*. Li and Racine [28] provide in-depth insight regarding the properties of these estimators.

## 5. Data

Malaria outbreak data were drawn from WHO reports [3,14] for 1990 to 2000 of 25 African countries representing a balanced panel with 319 observations. Associated monthly temperature and precipitation data were drawn from Mitchell *et al.* [38,39]. Data on demographic and other control variables were assembled as follows:

- Population and GDP data were drawn from the World Development Indicators [40] and CIA World Factbook [41].
- Data on the country specific gini inequality index and area in square kilometers per country (used to compute the population density) are obtained from the CIA World Factbook [41].
- Per capita expenditures on health were obtained from the WHO report [14].
- Data on the number of hospital beds per 1,000 people were obtained from the Organization for Economic Cooperation and Development [42].

The socio-economic variables were only available in the form of annual observations. Thus we aggregated our other data to an annual basis and for the climate variables computed variances using the monthly observations. As a result, all of our observed variables are in annual frequency. The key summary statistics of all the variables used in the estimation are given in Table 1.

The summary statistics indicate that there is generally less variability in temperature than in precipitation for most of the countries. Therefore, only variability in precipitation is used in the regression as a measure of climate variability index.

## 6. Empirical Model Specifications

Two specifications of the nonlinear function *f* are assumed and estimated. The first assumes an additive form and the second, a complex function of unknown form of *f*. In the first specification, the function *f* is smooth in the climate variability index measured by monthly standard deviation in precipitation. In such case, climate variability affects the number of malaria cases not only through the intercept (*θ*_0_) but also through the slope of temperature (*θ*_1_) and precipitation (*θ*_2_). That is, the effect of temperature and precipitation on the number of cases will depend on how precipitation variability evolves (consistent with the argument made by Jaenisch and Patz [25]). In other words, the size of the impact of temperature and precipitation on the malaria prevalence is conditioned by fluctuations in climate measured by the standard deviation in precipitation. The empirical statistical relationship between malaria cases, climatic and socio-economic variables can be written as:

(8) $$\begin{array}{l} \text{Log}({\text{CAPCASES}}_{\text{it}})=\theta_{0}({\text{STDPRECIP}}_{\text{it}})+{\text{TEMP}}_{\text{it}}*\theta_{1}({\text{STDPRECIP}}_{\text{it}})+\\ {\text{PRECIP}}_{\text{it}}*\theta_{2}({\text{STDPRECIP}}_{\text{it}})+\beta_{1}{\text{CAPGDP}}_{\text{it}}+\beta_{2}{\text{GINI}}_{\text{it}}+\beta_{3}{\text{POPDENS}}_{\text{it}}+\beta_{4}{\text{CAPEXP}}_{\text{it}}+\\ \beta 5\text{CAPBEDit}+\alpha i \end{array}$$

where *θ_l_*, for *l* = 0,1,2 are smooth coefficients (or functions of climate variability) and *β_k_* are the coefficients of the linear socio-economic control variables. Note that in this specification the interaction between temperature and precipitation is established through the variability in precipitation.

In the second specification where the form of the nonlinear function is unknown, the empirical formulation of the model can be written as:

(9) $$\begin{array}{l} \text{Log}({\text{CAPCASESE}}_{\text{it}})=f({\text{TEMP}}_{\text{it}},\;{\text{PRECIP}}_{\text{it}},\;{\text{STDPRECIP}}_{\text{it}})+\beta_{1}{\text{CAPGDP}}_{\text{it}}+\beta_{2}{\text{GINI}}_{\text{it}}+\\ \beta_{3}{\text{POPDENS}}_{\text{it}}+\beta_{4}{\text{CAPEXP}}_{\text{it}}+\beta_{5}{\text{CAPBED}}_{\text{it}}+\alpha_{i} \end{array}$$

Although, this last specification is the most attractive for empirical investigation proposes because it nests all possible functional forms of the effect of climatic factors on malaria prevalence, it comes with the cost that higher dimensionality of the function *f* might weaken the correct estimation of the marginal effects given the sample size in hand. Since we are fitting a three dimensional function with fewer data points (our current sample size is 305 observations) we can run into the risk of not having enough data points in some neighborhoods for a good fit. This problem is known as “*curse of dimensionality*” in non-parametric estimations [30]. That is, when more data become available (e.g., 500 observations at least) more general non-parametric models with fewer assumptions could be tested).

## 7. Results and Their Interpretation

Results obtained from the two model specifications are similar in many aspects but because of possible “*curse of dimensionality*” issues only results from the first specification are presented. We use the natural log of malaria cases per 1,000 people as dependent variable.

Results for the linear effects of the socio-economic variables on malaria cases are presented in Table 2. These effects are all statistically different from zero (except the effect of population density) and have expected signs. Also, the coefficients on these variables have the expected signs. An increase in GDP per capita and per capita expenditures on healthcare significantly reduce the number of malaria cases. In contrast, a higher Gini inequality index (meaning more unequal distribution of income) and count of hospital beds per 1,000 people lead to an increase in the number of reported malaria cases. We included number of hospital beds per 1,000 people for each country as a proxy for access to medical care. The assumption is that the number of hospital beds per 1,000 people indicates ability of an infected person to get to a health care facility and get treatment. Therefore, areas with more access to health care facilities are more likely to have cases reported and this needed to be controlled for. Note also that the socio-economic variables explain about 22% (*R*^2^ = 0.22) of the variations observed in the log of malaria cases per 1,000 people.

**Figure 1.**
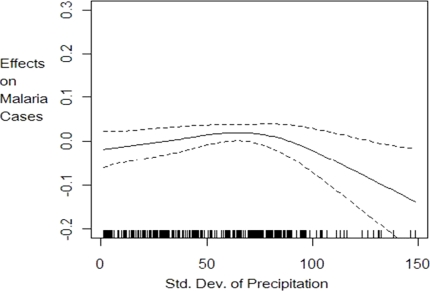
Effect of climate variability (standard deviation of precipitation) on per capita malaria cases with 95% confidence intervals. This graph is the plot of the function *θ*_0_(*STDPRECIP_it_*) in Equation (8). It describes the impact of climate variability measured as the standard deviation of precipitation on malaria prevalence.

**Figure 2.**
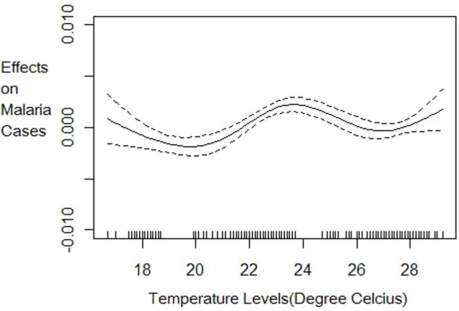
Effect of temperature levels on per capita malaria cases conditional on climate variability with 95% confidence intervals. This graph is the plot of the temperature function *TEMP_it_* * *θ*_1_ (*STDPRECIP_it_*) in Equation (8). It shows how temperature levels affect malaria prevalence given the current climate variability conditions measured as the standard deviation in precipitation.

**Figure 3.**
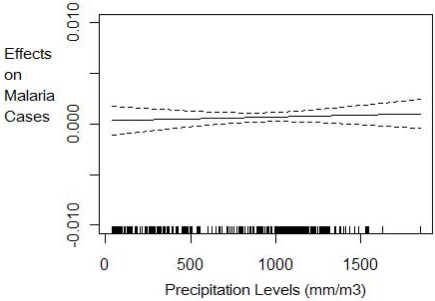
Effect of precipitation levels on per capita malaria cases conditional on climate variability with 95% confidence intervals. This graph is the plot of the precipitation function *PRECIP_it_* * *θ*_2_(*STDPRECIP_it_*) in Equation (8). It shows how temperature levels affect malaria prevalence given the current climate variability conditions measured as the standard deviation in precipitation.

The nonparametric component estimation results show that the three climate factors (temperature levels, precipitation levels and precipitation variability) explain 36% of the variation in the log of malaria cases. The 36% explanatory power of malaria cases by climate variables is lower than has been found in previous literature (e.g., Zhou *et al.*[7] and Ndiaye *et al.*[24]) likely because these previous models did not control for the relevant socio-economic confounding factors and included fewer countries. A nonlinear relationship is detected between malaria cases and climate variability and temperature but a linear constant effect of precipitation levels at any given climate variability realization. The effect of climatic factors on the number of malaria cases follows patterns shown in Figures 1 to 3. Lower climate variability (less than 0.19 standard deviations) has positive impact on malaria cases but as variability increases above 0.19 standard deviations, we observe a decrease in the number of cases but with high uncertainty, as shown by wider 95% confidence intervals (Figure 1).The effect of temperature on malaria cases at any given climate variability follows an increasing but not linear trend (Figure 2). Below 20 degrees the effect is negative and decreasing as expected since lower temperature levels do not permit survival of adult mosquitoes and therefore reduce their abundance. The effect is increasing for temperature levels between 20 ºC and 25 ºC but becomes positive only from 22 ºC as shown in Figure 2. At temperature levels above 25 ºC, the effect slows down but remains positive with an increasing trend. The pattern of the effect of temperature on malaria cases is consistent with the description given in MARA/ARMA report [12]. Note that since the range of temperatures in our sample is between 16.7 ºC and 29.2 ºC with the mean at 24.1 ºC we expect marginal increases in temperature to increase malaria prevalence in many of the countries of study. Precipitation levels have small but statistically significant effect on the number of cases (Figure 3). Also we conducted a nonparametric Fisher test [26,27,43] which fail to reject the hypothesis that all estimated coefficients are statistically different from zero at 1% significance level.

We tested several model specifications in which; (1) an interaction term of temperature and precipitation is included in the model, (2) three interaction terms resulting from combinations of the three climatic factors are included in the model, and (3) pure linear model specifications with or without interaction terms are estimated. Using a Bayesian model selection criterion (Akaike Information Criteria (AIC)) as in Woods [43], we find that nonparametric model specifications with or without temperature and precipitation interaction term perform the best and all the linear specifications perform poorly. However, the temperature and precipitation interaction term is statistically insignificant (in fact the interaction effects are already captured through the monthly standard deviation of precipitations variable).We retained the nonparametric model specification without temperature and precipitation interaction term.

The varying-coefficients obtained for temperature and precipitation are used to calculate the elasticity of the number of malaria cases with respect to climate factors for each of the 25 countries. Since the dependent variable is the log of malaria cases, the equation estimated is log (*y_it_*) = *f* (*X_it_*) + *βZ_it_*. The change in percentage of the number of malaria cases with respect to a one percent change in temperature or precipitation hereafter called elasticity is calculated as $e_{yx}=\frac{\partial y}{\partial x}\cdot \frac{x}{y}={\hat{f}}^{\prime }(x)e^{\left(\hat{f}(x)+\hat{\beta }z\right)}\cdot x/y$ and averaged over the 11 years period of study for each country.

The results in Table 3 (columns 3 and 4) suggest that the number of malaria cases in most of the countries is very responsive to changes in temperature excepting the findings in Botswana, Chad, South Africa and Tanzania where the response is small. Negative temperature effects are only observed in Central African Republic, Ethiopia and Guinea. The number of malaria cases is also very responsive to precipitation levels in Algeria, Central African Republic, Cote d’Ivoire, Djibouti, Egypt, Ethiopia, Ghana, Guinea, Malawi, Morocco, Rwanda, and Sudan but less responsive in Benin, Botswana, Burkina, Chad, Mauritania, Mali, Niger, South Africa, Togo, Uganda, Tanzania and Zimbabwe.

## 8. Effects of Climate to Date and Projections

The calculated elasticity is used to estimate both

- the consequences of recent climate change on the observed malaria cases to date and
- the effects of projected climate change in 2080 to 2099 to cases that would be observed under those conditions.

Between 1961 and 1990, the IPCC indicates average global temperatures have been increasing at the rate of 0.2 degree Celsius per decade [21] but precipitation has decreased by about 5–10 percent across the African continent [44]. We evaluated the effects of this on the estimated malaria cases using the elasticities estimated above. In particular we calculated for each country the change in temperature in degree Celsius and the percentage change in precipitation levels between the 1980–1989 decade and the 1990–2000 decade and used these values to estimate the effect of climate on malaria prevalence for these 20 years. The results are compiled in Table 3. These results show that excepting Benin, Burkina, Chad, Ethiopia, Ghana, Guinea, Sudan and Togo that all of the study region countries had an increase in the number of malaria cases due to climate change. The effect on the disease was particularly large in Algeria, Central African Republic, Djibouti, Egypt, Malawi and Morocco where we compute that recent climate change has contributed more than 21% increase in the number malaria of cases per 1,000 people.

The effects of future climate change on potential malaria cases was also estimated using projected temperature and precipitation alterations for 2100 drawn from 21 global climate models for the A1B emission scenario [21]. Temperature and precipitation projection values for Africa are not available at country level but rather for five larger regions (West, East, South, North and Mediterranean). The countries were assigned the projection for the region in which they fell. The potential changes in the number of malaria cases were computed at the minimum, median and maximum projected temperature/precipitation values corresponding respectively to scenario 1, scenario 2 and scenario 3 malaria cases projection in the Table 4. We found both positive and negative responses in the number of malaria cases depending on country and climate change scenarios. All but three countries showed a projected increase in the total number of cases across climate change scenarios. In the endemic zone around the equator the only countries to show a reduction across scenarios are Central African Republic, Ethiopia and Guinea. The general observation is that climate change is likely to enhance the number of cases in countries with high levels of currently infected people excepting a few countries. In particular, all other things held constant countries such as Algeria, Benin, Burkina, Burundi, Cote D’Ivoire, Djibouti, Egypt, Ghana, Malawi, Mauritania, Niger, Rwanda, Sudan, and Togo would see their malaria prevalence rate per 1,000 people increase more than 25% across climate change scenarios.

## 9. Estimated Cost of Treatment for Future Cases

The malaria outpatient and inpatient treatment cost is an economic burden for populations of endemic countries (all countries in this study are located in an endemic area except Algeria, Egypt, Morocco and South Africa). Outpatient treatment is related to treatments of mild cases using available malaria drugs without hospitalization for more than a day. However, for severe malaria cases (particularly for children under five) an inpatient treatment requiring hospitalization of several days is needed. The WHO recommends use of a line of drugs known as Artemisinin-based Combination Therapies (ACTs) although these are currently less accessible because of cost, demand and supply imbalance, and limited knowledge of safety [13]. We estimated the average prices of such drugs in Table 5 based on the reported drugs prices by the WHO [45] and the transportation costs (about 25% of the price) to reach the demand points [4]. The total outpatient costs (Table 6 column 3) for each individual country are calculated under minimal climate change projection scenarios (scenario 1). Similarly, average inpatient costs (Table 6 column 4) are calculated using information from studies conducted in Senegal and Kenya [46,47].

The cost projections indicate that the vast majority of the countries studied will see an increase of their costs of fighting the disease. We find that the predicted treatment costs, particularly the inpatient costs, as a percentage of 2000 health expenditure per 1,000 people, increase by more than 20% for countries such as Burundi, Cote D’Ivoire, Malawi, Rwanda and Sudan. Since most of the reported cases are severe cases (particularly children under five), the projected cost in malaria treatment will be most likely close to the inpatient treatment cost estimates.

## 10. Conclusions and Discussion

This paper studies the link between malaria cases and climatic factors in 25 African countries observed over 11 years. We conducted an econometric analysis and found that the number of malaria cases per 1,000 people is significantly influenced by climate factors in most of the countries studied, with disease incidence generally increasing under climate change. However, this effect is not uniform across countries as has been found in biophysical malaria modeling studies [16–18]. Additionally, the cost of malaria treatment is projected to increase in most of the countries studied.

Socio-economic factors are also found to impact significantly the number of malaria cases. In particular, we found that economic growth and better income distribution help reduce the number of malaria cases. Similarly, an increase in public health expenditures towards prevention and treatment of malaria significantly reduces malaria cases.

Thus policies that stimulate economic growth, reduce income inequality and increase public health expenditures would mitigate the impact of malaria. However, longer term climate policies are also desirable. These policies would include policies addressing greenhouse gas emissions reductions and those directed toward disseminating malaria related disease reduction practices plus research on eradication of malaria in Africa.

There are a few limitations to this study. Due to the lack of detailed meteorological information in the region, we assumed that all regions within each country are homogeneous and have same temperature and precipitation levels. The study also assumes that the disease response with respect to climatic factors will not change. Future research in understanding the impact of climate factors on malaria and other vector-borne disease may require more collaboration between biophysical scientists and economists.

Future research, could address economic growth, income distribution and changes in the level of public expenditure on healthcare in order to measure the impact of changes in socio-economic and disease control variables on projected malaria prevalence. This would extend work by Tol *et al.* [48] which investigates likely reduction of climate change impact on children mortality from various economic development scenarios using data for a fewer African countries. Also the use of spatial data on malaria such as those collected in MARA/ARMA project [12] would help provide more spatial details on specific countries.

## Figures and Tables

**Table 1.** Summary statistics.

**Variable**	**Definition**	**Mean**	**Std. Dev.**	**Min.**	**Max.**
CAPCASES	Malaria cases per 1,000 people.	95.2	119.64	0.0	947.4
TEMP	Temperature (Degree Celsius)	24.24	3.32	16.7	29.2
STDTEMP	Temperature standard deviation	2.9	1.9	0.3	8.5
PRECIP	Precipitation (mm/m^3^)	777.4	479.3	37	1,921.7
STDPRECIP	Precipitation standard deviation	60.0	41.1	1.3	220.4
POP	Population (million)	18.5	22.1	0.5	118.9
POPDENS	Population Density per km^2^	51.5	59.3	1.9	304.6
CAPGDP	Per capita GDP(US$/capita)	671.4	809.5	110.3	3,764.2
GINI	Gini inequality index	42.9	7.2	29.8	61
CAPEXP	Health expenditure ($/capita)	93.7	119.4	14	579
CAPBED	Hospital beds per 1,000 people.	1.2	0.9	0.1	4.8

**Table 2.** Linear coefficient estimates (b).

**Variables**	**Coefficients**	**Std. Dev.**	**T-Stats.**	**Other Stats.**
CAPGDP	−0.0008	0.0004	−1.86 **	
GINI	0.3721	0.0821	4.53 *	
POPDENS	0.0001	0.0047	0.03	
CAPEXP	−0.0266	0.0056	−4.71 *	
CAPBED	1.3648	0.4467	3.06 *	
CONSTANT	0.0321	0.0567	0.56	
Fisher-Stats (5,271)				12.87 *
R^2^				0.22
Hausman *χ*^2^ (5)-Stats (a)				13.24 *

* significant at 1% critical level;

** significant at 10% critical level.

(a) The significance of the Hausman *χ*^2^ statistic implies the rejection of the hypothesis of independence between the unobserved effects and the socio-economic variables. Therefore, the result presented in this table is obtained from fixed effects model specification.

(b) Since the dependent variable is the log of malaria cases per 1,000 people, the linear coefficients could not be directly interpreted as marginal effects but for any regressor *i* the marginal effect should be calculated as *β̂_i_* * *e^β̂Z^* by using logarithmic functions derivative rule.

**Table 3.** Estimated change in the number of malaria cases due to climate change in the past 20 years (a).

	Average annual cases per 1,000 people (1990–2000)	Cases Elasticity (%)		Computed change in number of cases per 1,000 people under under observed climate change past 20 years	Equivalent percentage change per 1,000 people
		to 1 º C change in Temp.	to 1% change in Precip.		
Algeria	0.01	155.25	2.38	0.00	0.33
Benin	86.53	23.93	−0.50	−8.81	−0.10
Botswana	31.05	1.78	−0.02	0.23	0.01
Burkina	60.99	19.92	−0.66	−3.99	−0.07
Burundi	168.53	14.30	0.16	2.17	0.01
Central Afr. Rep.	32.36	−27.73	10.89	10.32	0.32
Chad	45.14	0.87	−0.15	−0.12	0.00
Cote d'Ivoire	55.56	183.91	−13.69	8.25	0.15
Djibouti	9.73	143.36	4.63	16.67	1.71
Egypt	0.00	132.28	1.13	0.00	0.72
Ethiopia	6.19	−46.19	−84.68	−32.22	−5.21
Ghana	120.89	34.86	−1.85	−7.35	−0.06
Guinea	67.27	−12.44	−12.25	−62.18	−0.92
Malawi	381.81	10.47	−3.08	81.22	0.21
Mali	27.40	11.59	−0.01	0.64	0.02
Mauritania	62.29	21.94	0.31	4.35	0.07
Morocco	0.01	313.46	9.85	0.01	1.10
Niger	96.78	14.84	0.23	7.38	0.08
Rwanda	165.90	30.98	−2.90	2.54	0.02
South Africa	0.51	3.89	0.11	0.00	0.00
Sudan	228.75	29.16	−1.34	−18.33	−0.08
Togo	112.28	8.37	−0.23	−4.43	−0.04
Uganda	92.24	4.39	−0.32	2.23	0.02
Tanzania	302.68	1.97	0.00	1.19	0.00
Zimbabwe	98	12.65	−0.53	2.68	0.03

(a) The formula used to calculate the projected cases is based on the definition of elasticity. Let the elasticity value of the number malaria cases with respect to 1 °C change in temperature be *a*% and the elasticity value of the number of malaria cases with respect to 1% change in precipitation be *b*%. If the projected change in temperature in degree Celsius *c^o^* is and the projected change in precipitation is expressed in percentage as *d*%, then knowing the current number of malaria cases average *m*, the projected number of malaria cases is calculated as *p* = *m* × (*a* × *c* + *b* × *d*)/100.

**Table 4.** Projected cases change by the end of the century (2080–2100).

	Average annual cases per 1,000 people	Cases Elasticity (%)		Projected increase/decrease in cases per 1,000 people by the end of the Century (2080–2100) (a)		
	(1990–2000)	to 1 ºC change in Temp.	to 1% change in Precip.	Scenario 1	Scenario 2	Scenario 3
Algeria	0.01	155.25	2.38	0.02	0.05	0.07
Benin	86.53	23.93	−0.50	41.17	67.48	90.42
Botswana	31.05	1.78	−0.02	1.14	1.91	2.61
Burkina	60.99	19.92	−0.66	25.49	39.28	50.66
Burundi	168.53	14.30	0.16	42.55	79.01	110.41
Central Afr. Rep.	32.36	−27.73	10.89	−47.88	−22.56	14.23
Chad	45.14	0.87	−0.15	1.33	1.16	0.75
Cote d’Ivoire	55.56	183.91	−13.69	252.40	321.99	358.53
Djibouti	9.73	143.36	4.63	23.77	47.80	71.26
Egypt	0.00	132.28	1.13	0.01	0.01	0.01
Ethiopia	6.19	−46.19	−84.68	10.57	−45.82	−143.26
Ghana	120.89	34.86	−1.85	96.03	134.58	162.20
Guinea	67.27	−12.44	−12.25	59.12	−44.10	−171.21
Malawi	381.81	10.47	−3.08	217.00	182.96	121.42
Mali	27.40	11.59	−0.01	5.74	10.48	14.89
Mauritania	62.29	21.94	0.31	22.84	45.49	67.35
Morocco	0.01	313.46	9.85	0.04	0.10	0.16
Niger	96.78	14.84	0.23	23.88	47.85	71.05
Rwanda	165.90	30.98	−2.90	106.97	130.78	100.65
RSA	0.51	3.89	0.11	0.03	0.07	0.10
Sudan	228.75	29.16	−1.34	129.26	192.00	210.21
Togo	112.28	8.37	−0.23	19.25	30.49	40.00
Uganda	92.24	4.39	−0.32	8.17	10.88	10.00
Tanzania	302.68	1.97	0.00	10.73	19.15	25.81
Zimbabwe	97.53	12.65	−0.53	29.66	0.00	4.84

(a) The formula used to calculate the projected cases is based on the definition of elasticity. Let the elasticity value of the number malaria cases with respect to 1°C change in temperature be *a*% and the elasticity value of the number of malaria cases with respect to 1% change in precipitation be *b*%. If the projected change in temperature in degree Celsius is *c*° and the projected change in precipitation is expressed in percentage as *d*%, then knowing the current number of malaria cases average *m*, the projected number of malaria cases is calculated as *p*=*m* × (*a* × *c* + *b* × *d*)/100.

**Table 5.** Outpatient and inpatient unit treatment cost in 2004 USD.

	**Price($)**	**Test($)**	**Transport($)**	**Total($)**
*Drug prices*				
Artesunate	0.54	1.39	0.48	2.41
Artesunate-Mefloquine	0.36	1.39	0.44	2.18
Artemether-Lumefantrine	0.15	1.39	0.38	1.92
Artesunate-Amodiquine	0.08	1.39	0.37	1.83
**Mean outpatient cost**				**2.08**
*Hospitalization treatment cost*				
Kenya				64.00
Senegal				70.00
**Mean Inpatient cost**				**67.00**

**Table 6.** Estimated inpatient and outpatient treatment cost under climate change scenario 1.

	Projected cases per 1,000 people under Scenario1	Treatment costs per 1,000 people (in 2004 USD)		Treatment costs (in percentage of 2000 health expenditure per 1,000 people)	
		Outpatient(a)	Inpatient(b)	Outpatient (%)	Inpatient (%)
Algeria	0.02	0.05	1.63	0.0	0.0
Benin	41.17	85.76	2,758.58	0.3	8.1
Botswana	1.14	2.36	76.08	0.0	0.0
Burkina	25.49	53.08	1,707.60	0.1	3.2
Burundi	42.55	88.63	2,851.00	0.6	20.4
Central Afr. Rep.	−47.88	−99.73	−3,208.11	−0.2	−6.4
Chad	1.33	2.78	89.33	0.0	0.2
Cote d'Ivoire	252.40	525.71	16,911.11	0.7	21.4
Djibouti	23.77	49.50	1,592.37	0.1	2.5
Egypt	0.01	0.01	0.35	0.0	0.0
Ethiopia	10.57	22.02	708.40	0.1	3.7
Ghana	96.03	200.02	6,434.28	0.2	6.3
Guinea	59.12	123.14	3,961.17	0.2	5.2
Malawi	217.00	451.96	14,538.70	1.1	35.5
Mali	5.74	11.95	384.31	0.0	1.2
Mauritania	22.84	47.58	1,530.61	0.1	4.8
Morocco	0.04	0.09	2.87	0.0	0.0
Niger	23.88	49.73	1,599.67	0.2	6.4
Rwanda	106.97	222.79	7,166.67	0.7	22.4
South Africa	0.03	0.06	2.06	0.0	0.0
Sudan	129.26	269.22	8,660.18	0.7	21.7
Togo	19.25	40.10	1,289.89	0.1	2.6
Uganda	8.17	17.03	547.69	0.0	0.7
Tanzania	10.73	22.35	718.98	0.1	2.9
Zimbabwe	29.66	61.78	1,987.38	0.0	1.2

(a) Average outpatient treatment costs are calculated by multiplying the number of projected malaria cases by the average drug prices in Table 5.

(b) Average outpatient treatment costs are calculated by multiplying the number of projected malaria cases by the average hospitalization costs in Table 5.
